# A new Jun amino-terminal kinase inhibitor, KRev-202, inhibits rat ischemic acute injury and the progression to renal fibrosis

**DOI:** 10.3389/fphar.2025.1667221

**Published:** 2026-01-30

**Authors:** David J. Nikolic-Paterson, Greg H. Tesch, Elyce Ozols, Kurt Jarnagin, Yoshi Satoh, David R. Webb, Elizabeth Squiers, Keren Grynberg, Frank Y. Ma

**Affiliations:** 1 Department of Nephrology, Monash Medical Centre, Clayton, VIC, Australia; 2 Centre for Inflammatory Diseases, Monash University, Clayton, VIC, Australia; 3 Rev Therapeutics, San Diego, CA, United States

**Keywords:** acute kidney injury, CC-930, fibrosis, inflammation, ischemia, Jun amino-terminal kinase, KRev-202

## Abstract

**Introduction:**

Ischemia is an important cause of acute kidney injury (AKI). Ischemia-induced hypoxia rapidly induces activation of the Jun amino-terminal kinase (JNK) in tubular epithelial cells of the kidney, and blockade of this enzyme is protective in short-term animal models of renal ischemia. However, the clinical translation of this finding requires a water-soluble JNK inhibitor. This study investigated whether KRev-202, a soluble prodrug of the potent and selective JNK inhibitor CC930, can prevent ischemia-induced AKI and whether short-term inhibition of JNK can prevent AKI from transitioning to renal fibrosis.

**Methods:**

In a rat model of bilateral renal ischemia/reperfusion injury (IRI), the animals received prophylactic treatment with KRev-202, the parent compound (CC-930), or a vehicle by oral gavage, starting 1 h prior to surgery.

**Results and Discussion:**

In study 1, the animals were killed on day 1 after IRI to assess the AKI peak. Vehicle-treated animals exhibited a 4.5-fold increase in plasma creatinine levels, substantial tubular necrosis, increased tubular damage markers, and inflammation on day 1. Both KRev-202 and CC-930 treatment inhibited JNK activation, caused a 50% reduction in plasma creatinine levels, and substantially reduced tubular necrosis, tubular damage, and inflammation. In studies 2 and 3, treatments were administered from −1 h until day 4, and then the animals were killed on days 7 and 21, respectively. Compared to the vehicle group, a 4-day treatment with KRev-202 or CC-930 improved the recovery of tubular structure on day 7 and substantially reduced the development of renal fibrosis on day 21. Furthermore, KRev-202 treatment administered only during the first 24 h of IRI provided the same benefits as the 4-day treatment regimen, demonstrating the importance of early blockade of this pathway. In conclusion, KRev-202 is a new water-soluble JNK inhibitor with therapeutic potential for preventing ischemia-induced AKI.

## 1 Introduction

Acute kidney injury (AKI) is a clinical syndrome defined by an acute increase in serum creatinine levels or a severe reduction in urine output over 48 h (Makris and Spanou, 2016). AKI complicates 10%–15% of hospitalizations and is independently associated with increased mortality (Al-Jaghbeer et al., 2018). Ischemia is a common cause of AKI due to interruption of the blood supply to the kidneys (e.g., cardiac arrest and resuscitation and kidney transplantation) or due to a major decrease in mean arterial pressure (e.g., cardiac bypass surgery, hypovolemic shock, or sepsis) (Makris and Spanou, 2016; Kellum et al., 2021; Thompson et al., 2010). Severe AKI necessitates immediate dialysis and is associated with high mortality rates (Makris and Spanou, 2016; Al-Jaghbeer et al., 2018; Kellum et al., 2021). Those who survive an episode of AKI and recover their baseline kidney function remain at risk of progressing to advanced stages of chronic kidney disease (CKD) (Amdur et al., 2009; Ishani et al., 2009). AKI and CKD are now recognized as interconnected syndromes (Chawla et al., 2014).

The onset of AKI can be predicted in some settings. For example, AKI occurs in approximately 30% of patients undergoing cardiac bypass surgery (Kellum et al., 2021; Vives et al., 2014). This provides a direct opportunity for prophylactic treatment before kidney ischemia occurs to reduce the severity of AKI and the likelihood of AKI transitioning to CKD. However, despite many clinical trials, there are currently no approved specific therapies to prevent AKI during cardiac bypass surgery or in any other setting (Kellum et al., 2021).

Renal ischemia causes enormous changes in the kidney, with the induction of hypoxic and stress responses, acute metabolic dysfunction, and inflammation, which combine to cause tubular cell damage and death (Kellum et al., 2021). Jun amino-terminal kinase (JNK) is a key component of the cellular stress response, being activated within minutes in response to hypoxia, UV radiation, osmotic stress, cytokines, and damage-associated molecular patterns (DAMPs) (Grynberg et al., 2017). As the name suggests, JNK can phosphorylate serine 63 and 73 in the amino-terminal domain of the Jun protein; this enables Jun to dimerize with the Fos protein to form the transcription factor activator protein 1 (AP-1), resulting in the rapid transcription of a wide range of genes (Minden et al., 1994; Behrens et al., 1999; Davis, 2000). Biopsy studies have shown *de novo* Jun phosphorylation at Ser63 in many tubular epithelial cells in transplanted human kidneys at 15–20 min after reperfusion, establishing the rapid activation of JNK signaling following renal ischemia in the human kidney (Kanellis et al., 2010). Multiple studies have shown that various small-molecule JNK inhibitors, including SP600125, CC-401, and CC-930, can suppress acute renal failure and histologic damage in mouse and rat models of renal ischemia/reperfusion injury (IRI) when administered before surgery (Kanellis et al., 2010; Grynberg et al., 2021; Grynberg et al., 2022; Wang et al., 2007). Furthermore, global or tubular-specific deletion of *Jnk1/Mapk8* provides substantial protection from IRI-induced AKI (Grynberg et al., 2021).

A major challenge in translating these promising preclinical findings is the insolubility of current JNK inhibitors, such as CC-930 and CC-90001. Although this would not be an issue for a once-daily capsule for treatment of patients with a chronic disease, a water-soluble JNK inhibitor would be highly preferable for treatment before and after cardiac bypass surgery. This study describes the preclinical testing of a novel, water-soluble JNK inhibitor, known as KRev-202. The aims of the study were (i) to determine the capacity of KRev-202 to suppress IRI-induced AKI and to compare this with the current best JNK inhibitor, CC-930, and (ii) to determine whether short-term blockade of JNK signaling would suppress the induction of renal fibrosis as AKI transitions to CKD.

## 2 Materials and methods

### 2.1 Reagents and antibodies

CC-930 is a water-insoluble JNK inhibitor, which inhibits JNK1, JNK2, and JNK3 *in vitro*, with IC_50_ values of 0.061, 0.007, and 0.006 μM, respectively, and inhibits total JNK activity in a cell lysate assay, with an IC_50_ value of 0.2 μM (Plantevin Krenitsky et al., 2012). CC-930 has been used to inhibit JNK activity in animal models of kidney disease by oral gavage at 60 mg/kg b.i.d. (Grynberg et al., 2021; Yang et al., 2021; Lim et al., 2011). CC-930 was synthesized by WuXi AppTec (Tianjin, China).

KRev-202 is a water-soluble prodrug of CC-930, with a solubility of ∼49 mg/mL as measured at pH 7.4 in phosphate buffer. KRev-202 is converted to CC-930 *in vivo* and achieves comparable peak blood levels within 15 min of oral gavage at an equivalent molar dose with CC-930 in rats. KRev-202 was also synthesized by WuXi AppTec.

The primary antibodies used in this study were as follows: rabbit monoclonal anti-α-SMA/Acta2 (CS-19245) and rabbit monoclonal anti-collagen 1a1 (CS-72026) (Cell Signaling Technology, Danvers, MA, United States), along with goat polyclonal anti-KIM1/HACVR1 (AF 1817) (R&D Systems, Minneapolis, MN, United States). The secondary antibodies were biotin-conjugated goat anti-rabbit IgG (B8895, Sigma-Aldrich, St. Louis, MI, United States) and biotin-conjugated rabbit anti-goat IgG (A10518, Thermo Fisher Scientific, Waltham, MA, United States).

### 2.2 Animal model of renal ischemia and drug treatment

Outbred male Sprague–Dawley rats were obtained from the Monash Animal Research Platform (Clayton, VIC, Australia). Ten-week-old rats were anaesthetised with 75 mg/kg ketamine and 10 mg/kg xylazine and then placed on a heated blanket, which regulated body temperature to 37 °C using a rectal thermometer. A midline abdominal incision was made, and then, both renal pedicles were clamped for 25 min using non-traumatic vascular clamps. During this time, the abdomen was temporarily sutured to maintain body temperature and minimize fluid loss. After this time, clamps were removed, and the reperfusion of the kidneys was assessed visually. The wound was sutured in two layers, and saline was administered via subcutaneous injection. Postoperative pain relief consisted of a subcutaneous injection of buprenorphine (0.05 mg/kg) and 2–3 drops of bupivacaine onto the sutures at the end of surgery.

Study 1 (day 1): Four groups of six rats underwent renal IRI or sham surgery and were killed 24 h later by anaesthesia with 75 mg/kg ketamine and 10 mg/kg xylazine followed by exsanguination. The three groups undergoing renal IRI surgery received drug or vehicle treatment by oral gavage 1 h before surgery and 9 h after surgery (two doses in total). Drug treatment consisted of (i) KRev-202 at 104 mg/kg in a PBS vehicle, (ii) CC-930 at 60 mg/kg in 0.5% carboxymethylcellulose and 0.25% Tween-20 vehicle, or (iii) a PBS vehicle alone. Previous studies have shown that 60 mg/kg b.i.d. of CC-930 is effective in this rat IRI model (Grynberg et al., 2021). An additional group of six rats underwent sham surgery and were killed on day 1.

Study 2 (day 7): Three groups of six or seven rats underwent renal IRI surgery and were killed 7 days later. The three groups undergoing renal IRI surgery received drug or vehicle treatment by oral gavage 1 h before surgery, followed by eight additional doses administered b.i.d. over 4 days (nine doses in total). Drug dosing was determined based on the half-life of the compounds. Since the half-life of CC-930 in rat blood following p.o. dosing is approximately 14 h, a split b.i.d. dose of 60 and 30 mg/kg over 24 h was used to achieve plasma levels of CC-930 above 1,569 ng/mL for >18 h, but no more than 9,400 ng/mL at C_max_. KRev-202 is rapidly converted to CC-930 in circulation, and since KRev-202 has a half-life following p.o. dosing of approximately 6 h, 104 mg/kg b.i.d. dosing was used to achieve comparable blood levels to those of the CC-930 dosing. The groups of animals were treated with (i) KRev-202 at 104 mg/kg b.i.d. in a PBS vehicle, (ii) CC-930 alternating doses of 60 and 30 mg/kg b.i.d. in a 0.5% carboxymethylcellulose and 0.25% Tween-20 vehicle, or (iii) a PBS vehicle alone. A 150-μL blood sample was collected from the tail vein on days 1, 4, and 7 to measure renal function. A group of normal rats was also killed for use as a control.

Study 3 (day 21): Four groups of six rats underwent renal IRI surgery and were killed 21 days later. Three groups undergoing renal IRI surgery received drug treatment by oral gavage 1 h before surgery, followed by eight additional doses administered b.i.d. over 4 days (nine doses in total). Drug dosing was determined based on the half-life of the compounds. The groups were treated with (i) KRev-202 at 104 mg/kg b.i.d. in a PBS vehicle, (ii) CC-930 alternating doses of 60 and 30 mg/kg b.i.d. in a 0.5% carboxymethylcellulose and 0.25% Tween-20 vehicle, or (iii) a PBS vehicle alone. A fourth group received KRev-202 treatment 1 h before surgery and 9 h and 23 h after surgery (three doses in total), and the animals were then killed on day 21. A 150-μL blood sample was collected from the tail vein on days 1, 4, 7, and 21 to measure renal function. An additional group underwent sham surgery and was killed on day 21.

It should be noted that studies 2 and 3 were run concurrently and shared the same day-21 sham group as the control. The doses were selected to achieve a target CC-930 plasma concentration of >3.5 μM over 8 h and to approximately match the blood levels of CC-930.

Animal studies were approved and overseen by the local animal ethics committee under approval numbers MMCB/2022/32 and MMCB/2022/15. All animal studies were conducted in accordance with the Australian Code for the Care and Use of Animals for Scientific Purposes, 8th edition (updated in 2021).

### 2.3 Analysis of kidney function and histologic damage

Plasma creatinine levels and blood urea nitrogen (BUN) levels were measured using a Dupont ARL Analyzer (Wilmington, DE, United States) by the Department of Biochemistry at Monash Health.

Kidney tubular damage was assessed on 2-μm tissue sections stained with periodic acid-Schiff’s reagent and hematoxylin. The percentage of tubular cross-sections exhibiting damage in the outer medulla was scored in consecutive high-power (×400) fields with damage characterized as one or more of the following: loss of the brush border, nuclear loss, sloughing of cells into the lumen, marked tubular dilation, or tubular atrophy. Scoring was performed on blinded slides.

Kidney inflammation was assessed on 2-μm tissue sections stained with hematoxylin and eosin. The degrees of mononuclear and polymorphonuclear cell infiltration and red blood cell congestion were assessed in consecutive high-power (×400) fields of the outer medulla using the following scoring system: 0, normal; 1, mild; 2, moderate; 3, severe. Scoring was performed on blinded slides.

### 2.4 Immunohistochemistry staining and quantification

Immunostaining for Kim1/Havcr1 and phosphorylated Jun (p-Jun) was performed on 4-μm sections of formalin-fixed kidney tissue using antigen retrieval with 0.1 M sodium citrate, pH 6.0, and a three-layer avidin–biotin peroxidase complex staining technique, as previously described (Masaki et al., 2004). Immunostaining for α-smooth muscle actin (α-SMA/Acta2) and collagen 1 was performed on 4-μm sections of methylcarn-fixed kidney tissue.

The interstitial area of a-SMA and collagen 1 staining was quantified through image analysis. Digital images of the entire kidney cortex were taken at ×100 magnification and analyzed using Olympus cellSens Standard 1.18 software (Olympus Life Sciences, Hachioji, Japan). Medium and large vessels were excluded from the region of interest, and analysis was performed following adjustment of the threshold settings and was expressed as the percentage of the stained area. All slides and images were blinded.

### 2.5 Real-time PCR

The Ambion RiboPure Kit (Thermo Fisher Scientific) was used to isolate RNA from frozen kidney samples, which was then reverse-transcribed using random primers and the SuperScript III First-Strand Synthesis System (Thermo Fisher Scientific). The PCR was conducted on a StepOne Real-Time PCR system (Thermo Fisher Scientific) using TaqMan probes. All primers/probes were purchased from Thermo Fisher Scientific. All amplicons were normalized against the 18S internal control in each reaction, and the relative amount of mRNA was determined using the comparative cycle threshold (ΔCt) method. Samples from studies 2 and 3 were run on the same plate and shared the day-21 sham control.

### 2.6 Statistical analysis

Data are presented as the mean ± SD. Data were analyzed using one-way ANOVA with Tukey’s multiple comparison test for parametric data and Dunn’s multiple comparison test for non-parametric data.

## 3 Results

### 3.1 Study 1: Day 1 after renal ischemia/reperfusion injury

**FIGURE 1:**
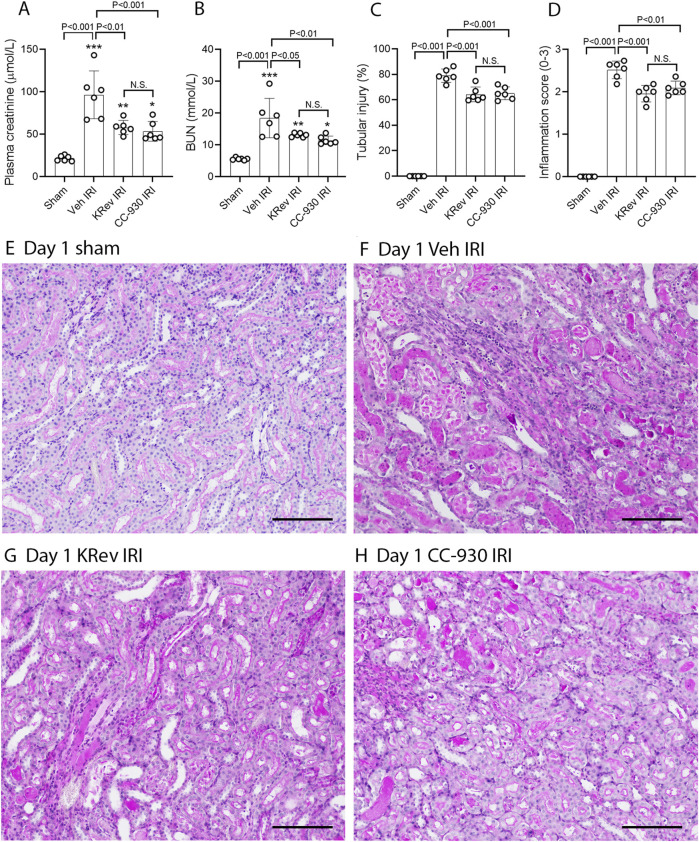
Renal function and histology on day 1 after IRI (Study 1). Plasma creatinine **(A)** and blood urea nitrogen (BUN) levels **(B)**. Scores of tubular injury **(C)** and kidney inflammation **(D)**. PAS staining of kidney tissues **(E–H)**, including the sham-operated control **(E)**, the vehicle-treated IRI **(F)**, the KRev-202-treated IRI **(G)**, and the CC-930-treated IRI groups **(H)**. Bars represent 200 μm. Data are presented as the mean ± S.D. ***p < 0.001 vs. the sham group; N.S., not significant.

**FIGURE 2:**
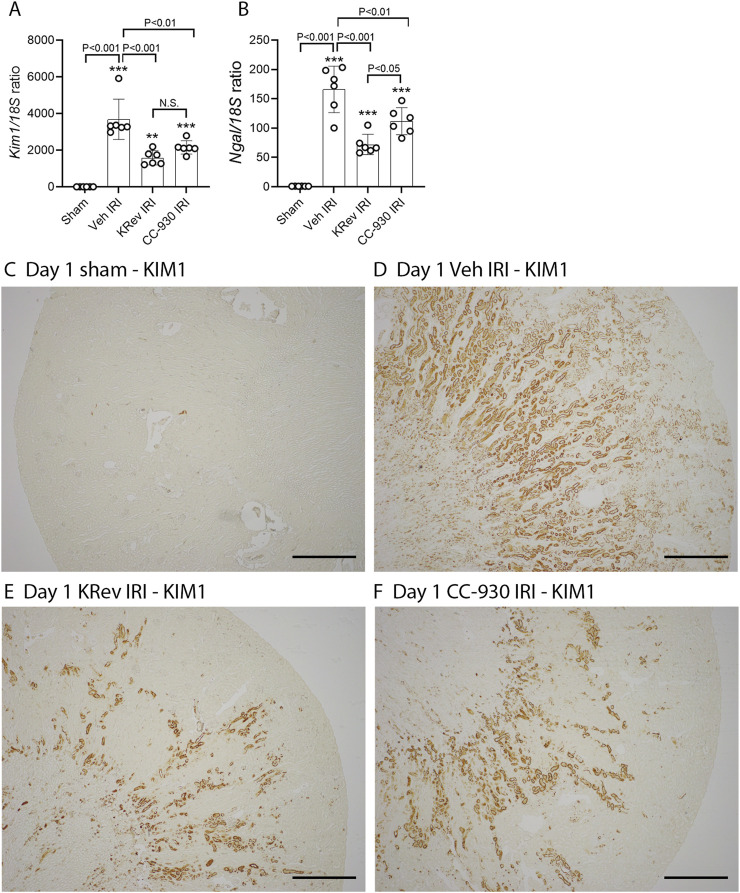
Tubular damage markers on day 1 after IRI (Study 1). Real-time PCR for *Kim1/Havcr1* **(A)** and *Ngal/Lcn2* mRNA levels **(B)**. Immunostaining for KIM1 **(C–F)**, including the sham control **(C)**, the vehicle-treated IRI **(D)**, the KRev-202-treated IRI **(E)**, and the CC-930-treated IRI groups **(F)**. Bars represent 700 μm. Data are presented as the mean ± S.D. **p < 0.01 and ***p < 0.001 vs. the sham group; N.S., not significant.

**FIGURE 3:**
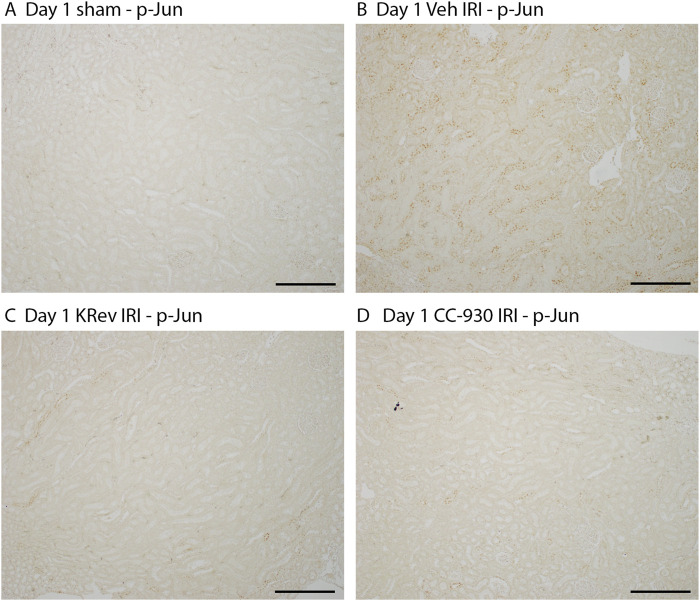
JNK activity on day 1 after IRI (Study 1). Immunostaining for phospho-Jun Ser63 in the sham control **(A)**, the vehicle-treated IRI **(B)**, the KRev-202-treated IRI **(C)**, and the CC-930-treated IRI groups **(D)**. Bars represent 200 μm.

To assess the potential of KRev-202 to prevent ischemia-induced acute kidney injury, animals were administered KRev-202, CC-930, or a vehicle alone 1 h before IRI surgery and again 9 h after surgery, and animals were killed 24 h after surgery. The vehicle-treated IRI group exhibited significant acute renal failure, as evidenced by a 4.5-fold increase in plasma creatinine levels and a 3.3-fold increase in BUN levels compared to sham controls (Figures 1A,B). This was accompanied by 80% of the tubular cross-sections in the outer medulla exhibiting damage, with prominent tubular necrosis and cast formation in the tubular lumen, which was absent in the sham controls (Figures 1C,E,F). Prominent inflammation (mononuclear and polymorphonuclear cell infiltration and red blood cell congestion) was evident in the vehicle-treated IRI group (Figure 1D). Consistent with the histologic damage, there was a dramatic increase in mRNA levels of tubular damage markers, *Kim1/Havcr1* and *Ngal/Lcn2*, in vehicle-treated animals (Figures 2A,B). Immunostaining showed widespread tubular staining of KIM1 in both the cortex and outer medulla of the vehicle-treated IRI group, which contrasted with the lack of staining in the sham control (Figures 2C,D). There was also marked *de novo* activation of the JNK pathway in the vehicle-treated IRI group, as shown by many tubular cells exhibiting nuclear staining for phospho-Jun Ser63, which was absent in the sham controls (Figures 3A,B).

Treatment with KRev-202 provided highly effective protection from AKI. There was a 50% reduction in the increase of plasma creatinine levels and a 40% reduction in BUN levels (Figures 1A,B). There was a clear reduction in the severity of tubular damage with KRev-202 treatment, including a reduction in the percentage of damaged tubules (Figures 1C,G) and a reduction in the inflammatory score (Figure 1D). There was also a substantial reduction in *Kim1* and *Ngal* mRNA levels with KRev-202 treatment, which was confirmed by a marked reduction in tubular immunostaining for KIM1 (Figures 2A,B,E). In addition, KRev-202 treatment largely abrogated Jun phosphorylation (Figure 3C), indicating effective inhibition of JNK signaling.

Treatment with CC-930 also significantly reduced ischemia-induced AKI. CC-930 was as effective as KRev-202 treatment in improving renal function, reducing histological damage, and inhibiting JNK activity (Figures 1–3). Of note, KRev-202 provided greater protection against the increase in *Ngal* mRNA levels than CC-930, and there was a non-significant trend toward lower *Kim1* mRNA levels with KRev-202 treatment compared with CC-930 treatment (Figures 2A,B).

### 3.2 Study 2: Day 7 after renal ischemia/reperfusion injury

The impact of JNK inhibition on the recovery from AKI was investigated in Study 2. The groups of animals were treated for 4 days with the vehicle, KRev-202, or CC-930 and then killed on day 7. The 4-day treatment period was selected, as this is generally the minimum length of hospital stay for the majority of patients undergoing cardiac bypass surgery .

**FIGURE 4:**
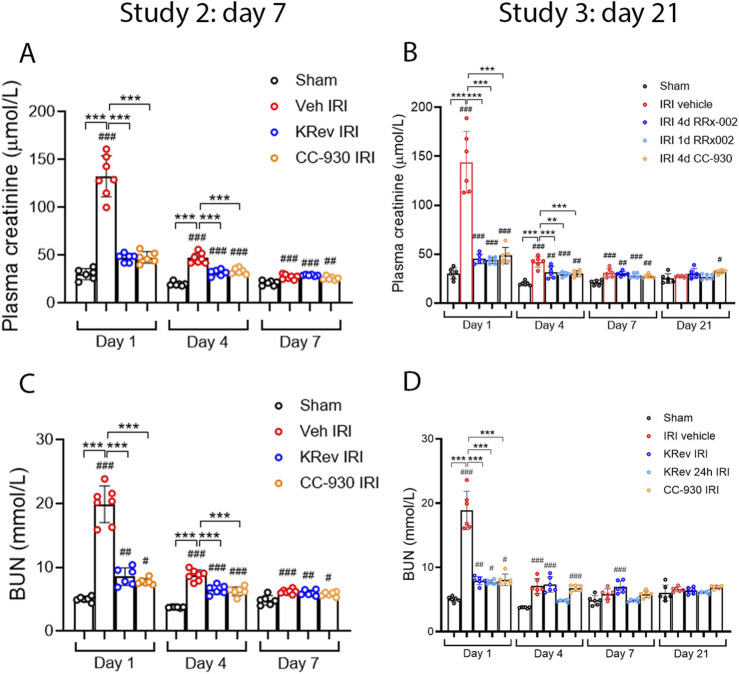
Renal function time course in Study 2 (day 7 after IRI) and Study 3 (day 21 after IRI). Plasma creatinine **(A,B)** and BUN levels **(C,D)**. Data are presented as the mean ± S.D. **p < 0.01 and ***p < 0.001. ^#^ *p* < 0.05, ^##^ *p* < 0.01, and ^###^ *p* < 0.001 vs. the sham group. N.S., not significant.

**FIGURE 5:**
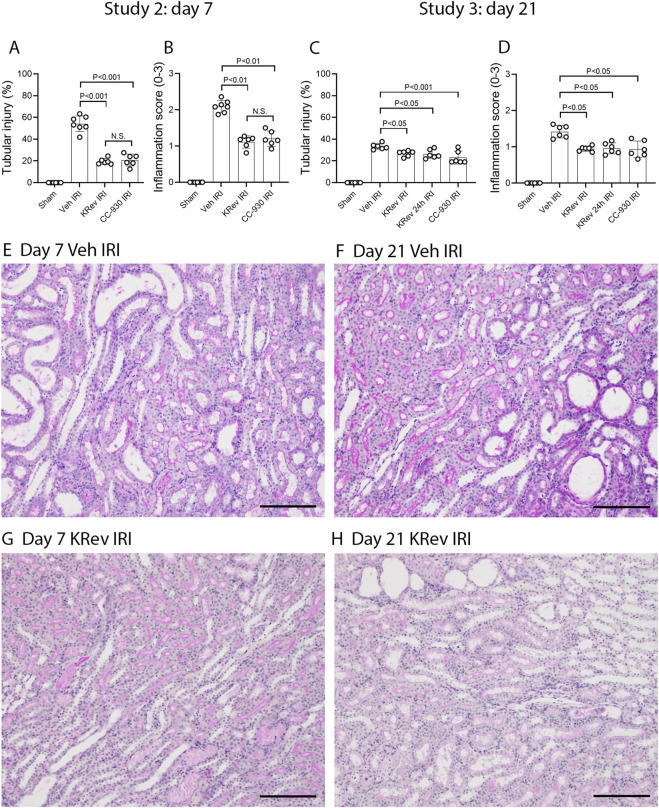
Renal histology in Study 2 (day 7 after IRI) and Study 3 (day 21 after IRI). Scores of tubular injury **(A,C)** and kidney inflammation **(B,D)**. PAS staining of kidney tissues **(E–H)**, including the vehicle-treated IRI group on day 7 **(E)** and day 21 **(F),** and the KRev-202-treated IRI group on day 7 **(G)** and day 21 **(H)**. Bars represent 200 μm. Data are presented as the mean ± S.D. N.S., not significant.

**FIGURE 6:**
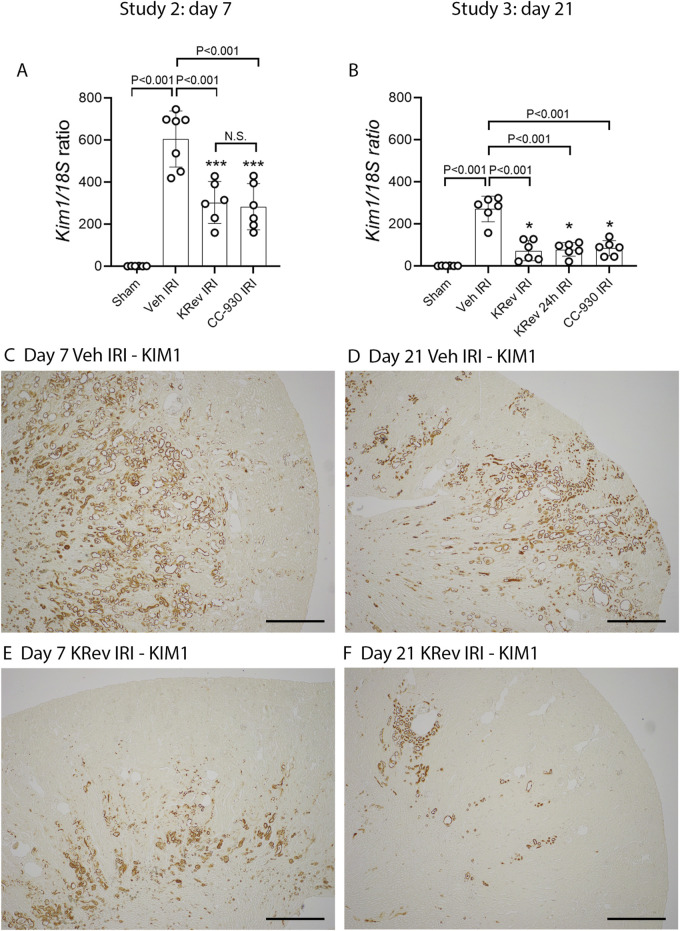
Tubular damage markers in Study 2 (day 7 after IRI) and Study 3 (day 21 after IRI). Real-time PCR for *Kim1/Havcr1* on day 7 **(A)** and day 21 **(B)**. Immunostaining for KIM1 **(C-F)**, including the vehicle-treated IRI group on day 7 **(C)** and day 21 **(D)** and the KRev-202-treated IRI group on day 7 **(E)** and day 21 **(F)**. Bars represent 700 μm. Data are presented as the mean ± S.D. *p < 0.05 and ***p < 0.001 vs. the sham group; N.S., not significant.

**FIGURE 7:**
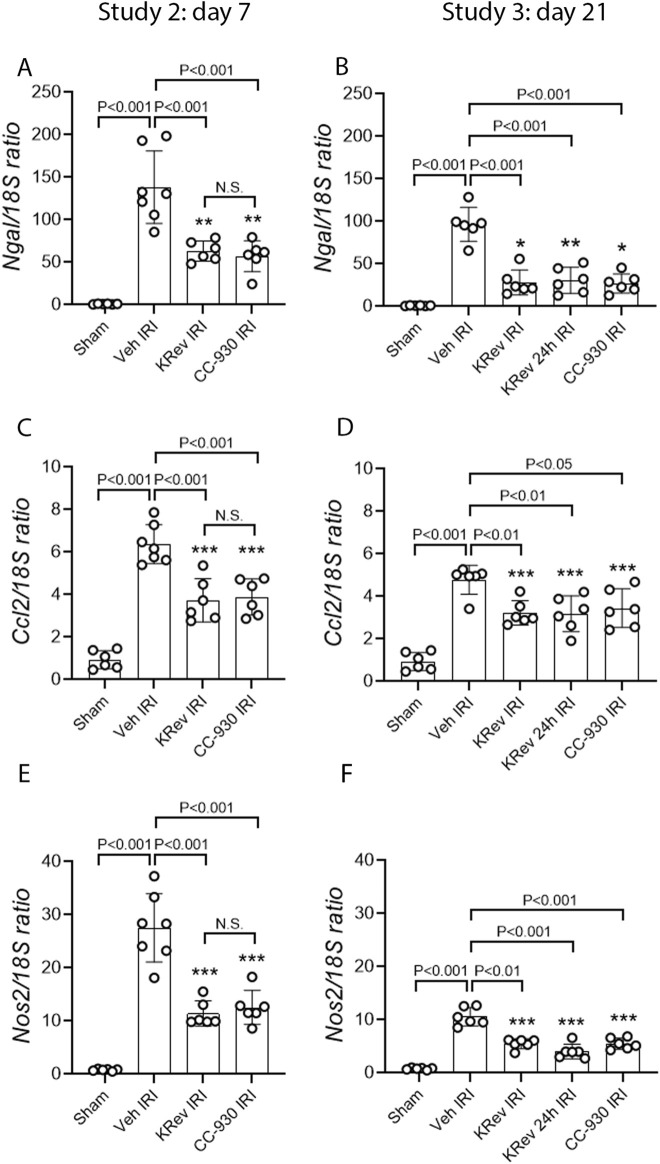
Tubular damage and inflammation markers in Study 2 (day 7 after IRI) and Study 3 (day 21 after IRI). Real-time PCR for *Ngal/Lcn2* on day 7 **(A)** and day 21 **(B)**; *Ccl2* on day 7 **(C)** and day 21 **(D)**, and *Nos2* on day 7 **(E)** and day 21 **(F)**. Data are presented as the mean ± S.D. *p < 0.05, **p < 0.01, and ***p < 0.001 vs. the sham group; N.S., not significant.

The vehicle-treated IRI group showed markedly elevated serum creatinine and BUN levels on day 1, which improved by day 4 and were near normal by day 7, although they remained above those of the sham control levels (Figures 4A,C). The percentage of injured tubules was reduced to 54% in the vehicle-treated IRI group on day 7 (Figure 5A). PAS staining showed focal areas of tubular damage in the outer medulla and inner cortex, featuring marked tubular dilation, some necrotic cells, and cellular debris in the tubular lumen. The remaining areas exhibited tubular cells recovering from damage, with re-establishment of normal tubular morphology, including the brush border (Figure 5E). Moderate cellular infiltration was still evident (Figure 5B), which was largely restricted to areas of tubular damage. Consistent with the histology, high levels of *Kim1* mRNA and staining of many tubules, including many dilated ones, were observed in the vehicle-treated IRI group on day 7 (Figures 6A,C). Elevated levels of *Ngal* mRNA were also evident on day 7 in the vehicle-treated IRI group (Figure 7A), and a significant inflammatory response was observed by increased *Ccl2* and *Nos2* mRNA levels (Figures 7C,E).

The 4-day treatment with KRev-202 provided marked protection against the increase in plasma creatinine and BUN levels on day 1 after IRI in the vehicle controls, and there were also lower plasma creatinine and BUN levels on day 4, but this protection was lost on day 7 (Figures 4A,C). Compared to the vehicle control, there was a marked improvement in renal histology with KRev-202 treatment on day 7 after IRI, with the majority of tubules showing recovery of normal structure and only 20% exhibiting injury, along with a reduced inflammation score (Figures 5A,B,G). Similarly, KRev-202 treatment substantially reduced *Kim1* and *Ngal* mRNA levels, with a considerable reduction in the area of KIM1 staining, along with a significant reduction in *Ccl2* and *Nos2* mRNA levels compared to the vehicle control on day 7 (Figures 6A,C, 7A,C,E). The 4-day treatment with CC-930 provided an equivalent protection from renal failure on day 1 and enhanced renal repair on day 7 after IRI, as that observed with KRev-202 treatment (Figures 4–7).

### 3.3 Study 3: Day 21 after renal ischemia/reperfusion injury

The impact of JNK inhibition on the AKI-to-CKD transition was investigated in Study 3. The groups of animals were treated for 4 days with vehicle, KRev-202, or CC-930 and then killed on day 21. In addition, one group was administered KRev-202 for only the first 24 h to assess whether a shorter period of JNK inhibition could provide comparable benefits to the 4-day treatment.

**FIGURE 8:**
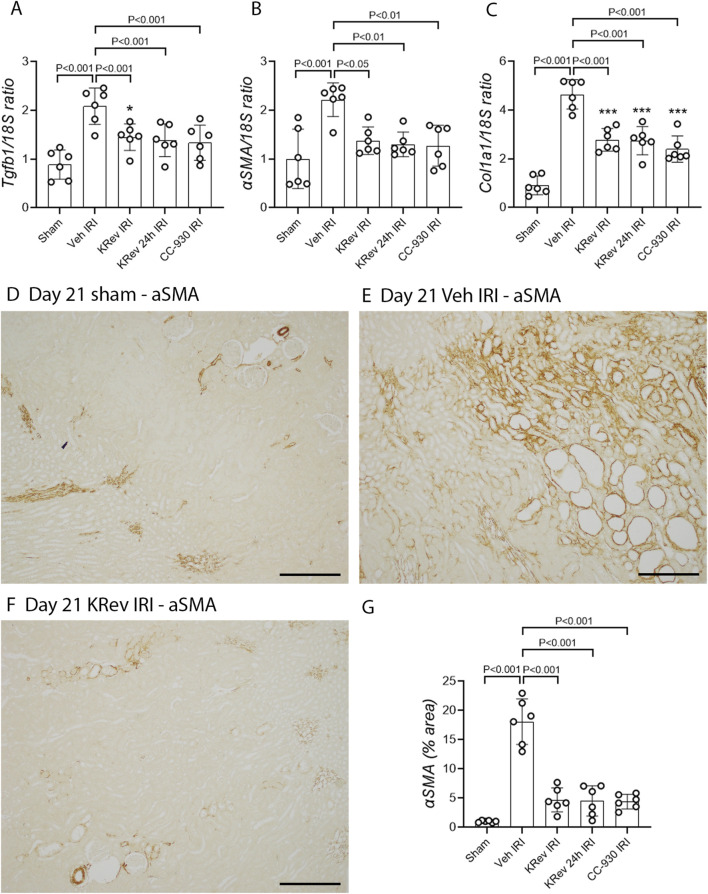
Fibrosis markers in Study 3 (day 21 after IRI). Real-time PCR for *Tgfb1* **(A)**, *αSMA/Acta2* **(B)**, and *Col1a1* **(C)**. Immunostaining for αSMA on day 21 in the sham control **(D)**, the vehicle-treated IRI **(E)**, and the KRev-202-treated IRI groups **(F)**. Area of interstitial collagen staining on day 21 after IRI **(G)**. Bars represent 200 μm. *p < 0.05 and ***p < 0.001 vs. the sham group; N.S., not significant.

**FIGURE 9:**
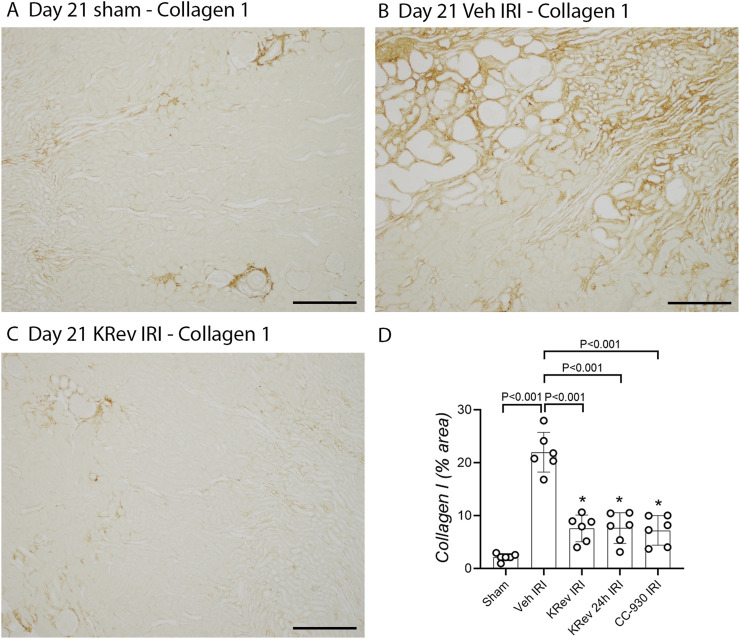
Collagen 1 deposition in Study 3 (day 21 after IRI). Immunostaining for collagen 1 on day 21 in the sham control **(A)**, the vehicle-treated IRI **(B)**, and the KRev-202-treated IRI **(C)** groups. Area of interstitial collagen staining on day 21 after IRI **(D)**. Bars represent 200 μm *p < 0.05. N.S., not significant.

The vehicle-treated IRI group showed markedly elevated serum creatinine and BUN levels on day 1, which improved by day 4 and returned to levels comparable to the sham control by day 21 (Figures 4B,D). The majority of tubules regained normal morphology by day 21; however, focal areas of dilated tubules were still evident, exhibiting atrophy, increased thickness of the tubular basement membrane, and an increased interstitial space around damaged tubules with numerous interstitial cells (Figures 5C,D,F). The incomplete repair in the vehicle-treated animals was clearly evident, with focal areas of tubules stained for KIM1 and persistently high levels of *Kim1* and *Ngal* mRNAs on day 21 (Figures 6B,D, 7B), while elevated levels of *Ccl2* and *Nos2* mRNA were also still evident on day 21 (Figures 7D,F). This incomplete repair was accompanied by significant renal fibrosis on day 21, with elevated mRNA levels of genes involved in fibrosis (Figures 8A–C), along with significant aSMA+ myofibroblast accumulation and collagen I deposition in the focal areas of tubular damage (Figures 8E,G, 9B,D).

The 4-day treatment with KRev-202 exerted a sustained benefit through day 21 after IRI. Consistent with the previous studies, KRev-202 treatment attenuated the acute increase in plasma creatinine and BUN levels observed on day 1 in the vehicle control IRI group, and creatinine levels also decreased more rapidly with KRev-202 treatment reaching the same levels as those in the sham control by day 21 (Figures 4B,D). In addition, KRev-202 treatment resulted in greater reductions in tubular injury and kidney inflammation scores on day 21 than the vehicle control group, with markedly improved histology observed on PAS staining (Figures 5C,D,H). Expression of tubular damage markers at both the mRNA and protein levels showed a marked improvement on day 21 with KRev-202 treatment (Figures 6B,F, 7B). Similarly, the expression of the inflammation markers *Ccl2* and *Nos2* was reduced with KRev-202 treatment compared to the vehicle control on day 21 (Figures 7D,G). Notably, renal fibrosis was substantially reduced by KRev-202 treatment, with reduced mRNA levels of fibrotic markers, along with a 79% reduction in aSMA+ myofibroblast accumulation and a 73% reduction in collagen I deposition (Figures 8, 9).

Of note, the 24-h treatment with KRev-202 provided the same degree of protection against IRI-induced renal functional impairment, histologic damage, activation of tubular injury markers, inflammatory responses, and renal fibrosis as the 4-day KRev-202 treatment (Figures 4–9).

Finally, the 4-day CC-930 treatment provided an equivalent protection against AKI and the transition to renal fibrosis as that observed with the 4-day or 24-h KRev-202 treatment (Figures 4–9).

## 4 Discussion

JNK signaling plays a key role in oxidant-induced tubular cell death (necroptosis and apoptosis) and inflammation, and JNK inhibition using small-molecule drugs or gene deletion can prevent ischemia-induced tubular cell damage and acute renal failure in rat and mouse models (Kanellis et al., 2010; Grynberg et al., 2021; Grynberg et al., 2022; Wang et al., 2007). In this study, a novel JNK inhibitor, KRev-202, was shown to provide substantial protection in a rat model of severe renal ischemia which transitions to renal fibrosis. Across all three studies, KRev-202 substantially reduced the peak of plasma creatinine and BUN levels on day 1 after IRI compared to the vehicle control, demonstrating reproducibility of the key finding. In addition, KRev-202 treatment was non-inferior to the JNK inhibitor, CC-930, demonstrating that the generation of CC-930 from its prodrug KRev-202 resulted in comparable inhibition of JNK activity in this animal model.

Activation of JNK signaling occurs within minutes of stress (Grynberg et al., 2017). In human kidney transplantation, JNK activation is evident in tubules at 15–20 min after reperfusion (Kanellis et al., 2010). In the rat IRI model, a dramatic induction of JNK activation is observed 30 min after reperfusion and decreases rapidly thereafter (Kanellis et al., 2010). Thus, it is evident that to prevent JNK-driven AKI, it is critical to have the JNK inhibitor onboard prior to the ischemic insult. Delaying treatment with a JNK inhibitor until 1 h after kidney reperfusion did not change the severity of IRI-induced AKI at the 24-h time point (Kanellis et al., 2010).

An important question is whether short-term, prophylactic JNK inhibition to reduce ischemia-induced AKI will affect the potential for subsequent progression to CKD. Clinical data indicate that the severity of AKI predicts progression to CKD (Chawla et al., 2011), implying that any reduction in the severity of AKI will reduce the subsequent transition to CKD. It is now recognized that maladaptive repair following AKI is an important contributor to the AKI-to-CKD transition (Yu and Bonventre, 2020). This occurs via the arrest of proliferating proximal tubular cells at the G2/M phase of the cell cycle, which activates JNK signaling and the subsequent production of pro-fibrotic cytokines, thereby promoting renal fibrosis. Thus, in this study, the ability of a 4-day period of prophylactic JNK inhibition to both significantly improve kidney repair on day 7 after ischemic injury and to substantially reduce the induction of renal fibrosis on day 21 may reflect both the reduced severity of the initial tubular damage and a reduction in proximal tubular cell G2/M arrest-driven fibrosis, although the latter mechanism was not assessed in the present study.

A limitation of our study is that we did not extend our observations beyond the 21-day period to determine whether targeting JNK activation during the early stages of renal ischemia provides long-lasting benefits. One study addressed this question by administering the JNK inhibitor, SP600125, at 24 h and 2 h before surgery and 12 h after surgery in a mouse IRI model (Liang et al., 2022). This was a very severe model, with a 17-fold increase in serum creatinine at 24 h in the vehicle-treated group, which was reduced by 30% with SP600125 treatment. Significant increases in *Col4a1* and *Fn* mRNA levels were observed at 4 weeks after IRI in the vehicle group, which progressively increased at 8 and 12 weeks. The SP600125-treated group showed significantly lower *Col4a1* and *Fn* mRNA levels at all time points, but there was still a trend of increasing fibrosis over 4–12 weeks following early JNK inhibitor treatment (Liang et al., 2022). Given that prophylactic JNK inhibitor treatment reduces renal fibrosis in obstructed rat kidneys and that intervention with CC-930 suppresses renal fibrosis in a mouse model of folic acid-induced AKI-to-CKD transition (Jiang et al., 2019; Ma et al., 2007), prophylactic plus ongoing JNK inhibitor treatment may be required to both limit AKI and prevent a slow transition to renal fibrosis and CKD.

Of relevance to the current experimental study design, administration of CC-930 in healthy volunteers at doses up to 200 mg/day over 6 days did not cause significant adverse effects, although a phase 2 study of patients with idiopathic pulmonary fibrosis reported elevated hepatic transaminases after 8–12 weeks at the highest dose of CC-930 (100 mg DIB), which resolved upon discontinuation of the drug (van der Velden et al., 2016). However, there are strategies to prevent JNK activation other than directly targeting JNK. Apoptosis signal-regulating kinase 1 (ASK1/MAP3K5), which is activated by oxidative stress, leads to the activation of both JNK and the p38 mitogen-activated protein kinase (Tesch et al., 2020). Mice lacking the *Ask1 gene* or prophylactic administration of an ASK1 inhibitor suppressed JNK activation and provided substantial protection against the acute loss of renal function in IRI mouse and rat models (Liles et al., 2018; Terada et al., 2007). In addition, late intervention with an ASK1 inhibitor suppressed cellular senescence and renal fibrosis in a model of renal IRI in diabetic mice (Tesch et al., 2024). Although ASK1 inhibitor treatment with selonsertib exhibits a good safety profile in clinical trials, a limitation of its use to prevent AKI is that it blocks tubular creatinine secretion, causing an artificial increase in serum creatinine levels (Chertow et al., 2019). In addition, a recent Phase 2b run-in trial of selonsertib in patients with diabetic kidney disease reported a numerically higher rate of investigator-reported AKI in those taking the drug (Heerspink et al., 2024), although it remains unclear whether this was related to the inhibition of p38 MAPK.

In summary, this study identified a novel, water-soluble JNK inhibitor, which has the potential for intravenous administration prior to cardiac bypass surgery to prevent or reduce ischemia-induced AKI and subsequent progression to CKD.

## Data Availability

The raw data supporting the conclusions of this article will be made available by the authors, without undue reservation.

## In memoriam

Dr Frank Y Ma and Dr Kurt Jarnagin died after the study was completed and the manuscript drafted.
